# Electrochemical Synthesis of Reduced Graphene Oxide/Gold Nanoparticles in a Single Step for Carbaryl Detection in Water

**DOI:** 10.3390/s22145251

**Published:** 2022-07-13

**Authors:** Ibtihaj Albalawi, Hanan Alatawi, Samia Alsefri, Eric Moore

**Affiliations:** Sensing and Separation Group, School of Chemistry, University College Cork, T12 YN60 Cork, Ireland; 110125574@umail.ucc.ie (I.A.); 110122737@umail.ucc.ie (H.A.); 112220405@umail.ucc.ie (S.A.)

**Keywords:** in situ synthesis approach, screen-printed carbon electrode, carbaryl–phenol, reduced graphene oxide (rGO), gold nanoparticles (AuNPs), environmental water

## Abstract

In this study, an in situ synthesis approach based on electrochemical reduction and ion exchange was employed to detect carbaryl species using a disposable, screen-printed carbon electrode fabricated with nanocomposite materials. Reduced graphene oxide (rGO) was used to create a larger electrode surface and more active sites. Gold nanoparticles (AuNPs,) were incorporated to accelerate electron transfer and enhance sensitivity. A cation exchange Nafion polymer was used to enable the adhesion of rGO and AuNPs to the electrode surface and speed up ion exchange. Cyclic voltammetry (CV), energy dispersive X-ray spectroscopy (EDX), X-ray photoelectron spectroscopy (*XPS*), electrical impedance spectroscopy (EIS), atomic force microscopy (AFM) and scanning electron microscopy (SEM) were performed to study the electrochemical and physical properties of the modified sensor. In the presence of differential pulse voltammetry (DPV), an rGO/AuNP/Nafion-modified electrode was effectively used to measure the carbaryl concentration in river and tap water samples. The developed sensor exhibited superior electrochemical performance in terms of reproducibility, stability, efficiency and selectivity for carbaryl detection with a detection limit of 0.2 µM and a concentration range between 0.5µM and 250 µM. The proposed approach was compared to capillary electrophoresis with ultraviolet detection (CE-UV).

## 1. Introduction

Pesticide use in grains is extensively reported, and this trend is likely to expand significantly over the next few decades [1]. According to a World Health Organization (WHO) report, roughly 87,000 cases of cancer that occur each year in poor nations are linked to pesticide use [2]. Among pesticides, carbamate compounds, which have great insecticidal activity, are the most extensively used pesticides in agriculture [3]. Carbaryl (C_12_H_11_NO_2_) is such a carbamate, which is frequently used in grains and is the second most common pesticide found in water. These toxic pesticides reach the human body via drinking water or the food chain and are rapidly absorbed and metabolized in the gastrointestinal system and, thus, pose a threat to human health due to their toxicity to the enzyme acetylcholinesterase (AChE), which is necessary for the healthy functioning of the human central nervous system [1,4]. Therefore, the sensitive, precise and quick detection of these carbamate pesticides is critical for environmental and human health protection.

Chromatography is frequently used to identify the presence of insecticides such as carbaryl in water and soil samples. Among the most often and recently used types of chromatography are chromatography coupled to mass spectroscopy [5,6], gas chromatography (GC) [7], high-performance liquid chromatography (HPLC) [8] and capillary electrophoresis (CE) [9]. However, there are several limitations to these methods, including: they are time-consuming; significant quantities of solvents are consumed; and precise extraction and cleaning procedures are required, making them complex and consequently inadequate for routine field operation. On the other hand, electrochemical sensors have the advantages of low cost, ease of operation, rapid response and great sensitivity.

**Figure 1:**
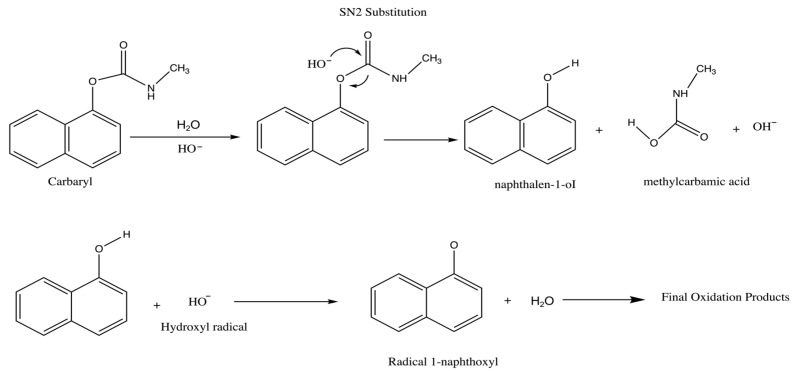
Scheme of the hydrolysis of carbaryl.

Enzyme-coupled materials have been frequently reported to increase the sensitivity and selectivity of electrochemical sensors for carbaryl detection. However, enzymatic-based assays are expensive and require a qualified scientist to use the instrument [10]. Moreover, enzymes employed as sensors require specific handling in terms of storage, temperature, enzyme activity, pH and applied potential, which makes these devices more complicated to deal with and less consistent in terms of reliability [11]. Recently, non-enzymatic methods have been reported which require the hydrolysis of carbaryl to carbaryl–phenol (Figure 1) to enhance the electrochemical reaction [12,13]. However, it was reported that carbamate residues can be detected directly, electrochemically, without the requirement for the target analytes to be first hydrolyzed [14,15,16]. The comparatively high detection potential required for these herbicides has a significant impact on sensitivity and selectivity. In previous studies, carbamate-hydrolyzed derivatives had a significantly lower oxidation potential and a favorable electrochemical performance, reducing interference and significantly increasing electrode efficiency.

However, due to the fast polymerization of electrogenerated phenoxy radicals, phenol derivatives cause electrode fouling during oxidization. The generated passive layer is strongly attached to the electrode surface. As a result, a time-consuming cleaning method is required [10]. Therefore, a disposable, screen-printed carbon electrode (SPCE) is an ideal alternative option, which has the characteristics of being disposable, affordable and easy to fabricate. Moreover, after each measurement, a single-use SPCE can be quickly and simply changed.

Carbon-based nanoparticles are among the most compatible nanomaterials and are extremely absorbent and have high electron transfer rates [17]. In the last five years, 0D–3D carbon allotropes have created great opportunities and considerable advances in electrochemical analysis. All allotrope forms of carbon, including graphite, diamond and fullerenes, are applied as electrode-based materials in modern chemistry [18]. Fullerenes and carbon nanotubes have attracted considerable attention, especially CNTs, which are widely applied in sensing applications due to their electrochemical properties and their unusual structures [19,20]. Diamond films have significant potential in electrochemical application due to their resistance and stability [21]. Carbon allotropes include graphene, a 2D, thin-layer structure which is the basic building block of carbon allotropes, and nanomaterials containing oxygenated, hydrophilic functional groups [22]. It has fascinating features, which include high mobility, huge surface areas, superior thermal conductivity, mechanical flexibility, chemical inertness and low cost [23,24]. Additionally, graphene-based materials have attracted considerable attention in new-generation devices for nanostructured electrodes [25,26]. The charge transfer interaction between analytes and the surface of the working electrode is critical for voltammetry sensing applications, and, therefore, controlling the electron transfer (ET) active sites on the graphene sheet is essential and can be adjusted by adding impurities and functional groups [27,28]. In comparison with graphene sheets, graphene oxide has been recently discovered and used as a novel electrode material due to its biocompatibility, affordability and simplicity of synthesis [29].

Recently, metal nanoparticle dispersion on graphene sheets has been widely recognized, which also creates a new route for the development of novel magnetic, optoelectronic and catalytic materials [30]. There are several methods to synthesize graphene-based metal nanocomposites, including sol–gel [31], solution mixing [32], hydrothermal [33], microwave irradiation [34], self-assembly [35] and electrochemical deposition methods [36]. Electrochemical deposition is a process for producing a layer of solid metal from an ion solution on a surface that is electrically conductive. This low-cost and efficient process has a number of benefits, including high purity of the components that are deposited and strict composition control [37]. Among the various metal nanoparticles, gold nanoparticles (AuNPs) have strong catalytic activity, excellent biocompatibility and a high rate of electron transfer [38]. The in situ synthesis of AuNPs onto reduced graphene oxide (rGO) is a promising strategy, producing huge surface ratios and unique binding sites on the electrode surface. Graphene and AuNPs are also suitable materials for a wide variety of applications due to their environmental friendliness and safety [39].

In terms of carbamate pesticides, graphene oxide (GO) and gold nanoparticles (AuNPs) were synthesized by A. Jirasirichote et al. using the Hummers and Turkevich methods to detect carbofuran–phenol in agricultural fields [40]. Despite its accurate and precise methods, the efficiency of reagent mixing and the resulting concentration gradient is a significant challenge in this field. Therefore, in this study, the in situ synthesis of AuNPs onto an rGO-modified, screen-printed carbon electrode based on electrochemical reduction was first studied to detect carbaryl–phenol in water samples. As a membrane matrix, Nafion (NA) has been utilized to improve electrode stability and speed up ion transfer. Additionally, reduced graphene oxide produces a great electrode surface area and a high number of active sites, while the incorporation of AuNPs accelerates electron transfer. The purpose of this work was to establish a simple and efficient electrochemical approach for carbaryl species determination in a variety of water samples. Differential pulse voltammetry (DPV) was performed to detected carbaryl–phenol, and the electrode surface was modified with nanomaterials to enhance the sensitivity and reduce the oxidation response time.

## 2. Experimental

### 2.1. Chemicals and Materials

All chemicals were used as analytic reagents and without additional purification. Deionized water (18.2 M cm specific resistance) was obtained from the laboratory water system at UCC (University College Cork). Graphene oxide solution (4 mg/2 mL), gold (III)chloride trihydrate, Nafion (5 wt% in a mixture of lower aliphatic alcohols and 45% water), carbaryl, acetic acid, phosphoric acid, sodium tetraborate and sodium acetate were purchased from Sigma Co., Ltd. (Dublin, Ireland). All experiments were performed at room temperature. A screen-printed carbon electrode was received from DropSens (Asturias, Spain). It contained a carbon working electrode with a diameter of 4 mm. The total size of the SPCE was L33 × W10 × H0.5 mm.

### 2.2. Solutions Preparation 

The hydrolysis of carbaryl-to-carbaryl phenol was achieved by dissolving 0.022 g of carbaryl in 100 mL of 0.1 M NaOH. To obtain the required hydrolysis, the standard solution was heated for 30 min at 70 °C. Acidic solution buffer was prepared by dissolving an appropriate amount of sodium acetate in 0.1 M acetic acid to adjust the pH to 5.

### 2.3. Nanocomposite-Modified Electrode Preparation

To form homogeneous suspensions, GO solution (2 mg/mL) was dispersed into deionized water and ultrasonicated for 15 min. After that, an aquatic solution of 5 mM HAuCl4 was added and stirred for 5 to 10 min. Then, Nafion (0.5 wt%) was diluted in absolute ethanol and sonicated for 10 min and added to the previous solution. Finally, the combination containing deionized water and ethanol in a 5:7 (*v*/*v*) ratio was stored in a dark environment. Prior to use, a disposable, screen-printed carbon electrode was activated by cycling the electrode between 0 and 1.6 for 3 scans in 0.5 M sulfuric acid solution. The electrode was then cleaned with ultrapure water and allowed to dry at air temperature. The treated carbon electrode was coated with a 6 µL suspension of GO, HAuCl4 and Nafion and kept at room temperature for 15 min to dry. Then, the electrode was cycled between −1.3 and +0.8 V in 0.5 M NaCl at 0.05 V/s for 6 potential cycles to reduce the suspensions. After the electro-reduction, the electrode was rinsed with ultrapure water and dried in air. In the same way, the rGO-modified electrode was generated.

### 2.4. Physical and Electrochemical Analysis

Palm Sens (Houten, The Netherlands) handheld potentiostat/galvanostat and CH instrument was used to investigate the performance of electrochemical analysis. The experiment was carried out using differential pulse voltammetry (DPV) in 0.1 M acetate buffer (pH = 5), and the voltammogram was recorded between −0.1 and 0.4 V under the following parameters: pulse potential, 0.05 V; pulse time, 0.2 s; amplitude, 0.13 V; a 0.01 V/s scan rate; and an equilibrium time of 2 s. Additionally, electrical impedance spectroscopy (EIS) and cyclic voltammetry (CV) were conducted in the presence of 1 mM K_3_[Fe(CN)_6_] in a 0.1 M of KCl solution for the purpose of characterization. Scanning electron microscopy (SEM), atomic force microscopy (AFM), UV–vis spectrophotometry and energy dispersive X-ray spectroscopy (EDX) were applied to determine the morphological characteristics of the modified nanocomposite electrode.

### 2.5. Real Sample Preparation

River water samples were collected from the Lee River (Cork, Ireland), and a membrane filter (0.22 µm) was used to remove any suspended material. Prior to analysis, a specific amount of sodium acetate and acetic acid was added to modify the pH of water samples to 5.

### 2.6. Traditional Capillary Electrophoresis with UV Detector (CE-UV)

Agilent CE 7100 (Waldbronn, Germany) with a UV detector was used with 50 µm internal diameter and a 375 µmod fused silica capillary 40 cm in length with an effective length of 31.5 cm (Composite Metal Services Ltd., Shipley, BD17 7AD, UK) under an applied voltage of 20 kV at 214 nm. The samples were injected hydrodynamically for 5 s at 50 mbar, and 50 mM sodium tetraborate buffer solution (pH 9.2) was used for separation.

### 2.7. Sample Preparation and Solid Phase Extraction (SPE)

River water samples were collected and filtered using a 0.22 µm membrane filter. For water analysis, 2.5 mL carbaryl stock solution was spiked into 7.5 mL river and drinking waters. The samples were extracted using a Sep-Pak C18 3 cc column (Waters, Ireland). Prior to column preconditioning, 6 mL deionized water and 3 mL methanol were used. Then, the spiked samples were loaded. To ensure optimal retention, the spiked samples were loaded at a consistent flow rate of 1 drop/s. After passing the spiked samples through the column, the column was dried for 2 min with nitrogen. The analytes were then released by adding 1 mL methanol to the column. In the same way, the blank was prepared and loaded with 10 mL sample water instead of the spiked samples. The eluents from the spiked samples and blank were diluted 25 times using running buffer [41].

## 3. Results and Discussion

### 3.1. Electrode Activation

**Figure 2:**
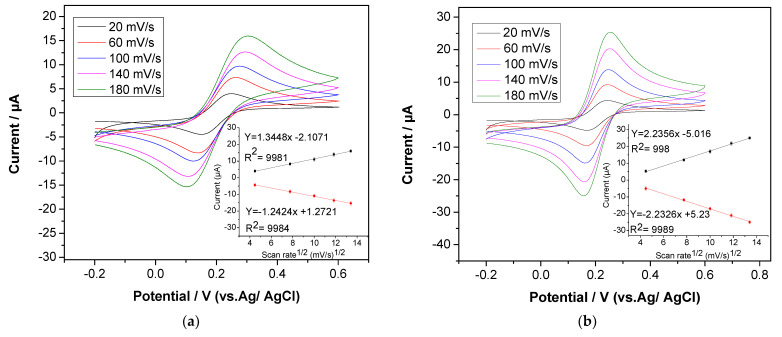

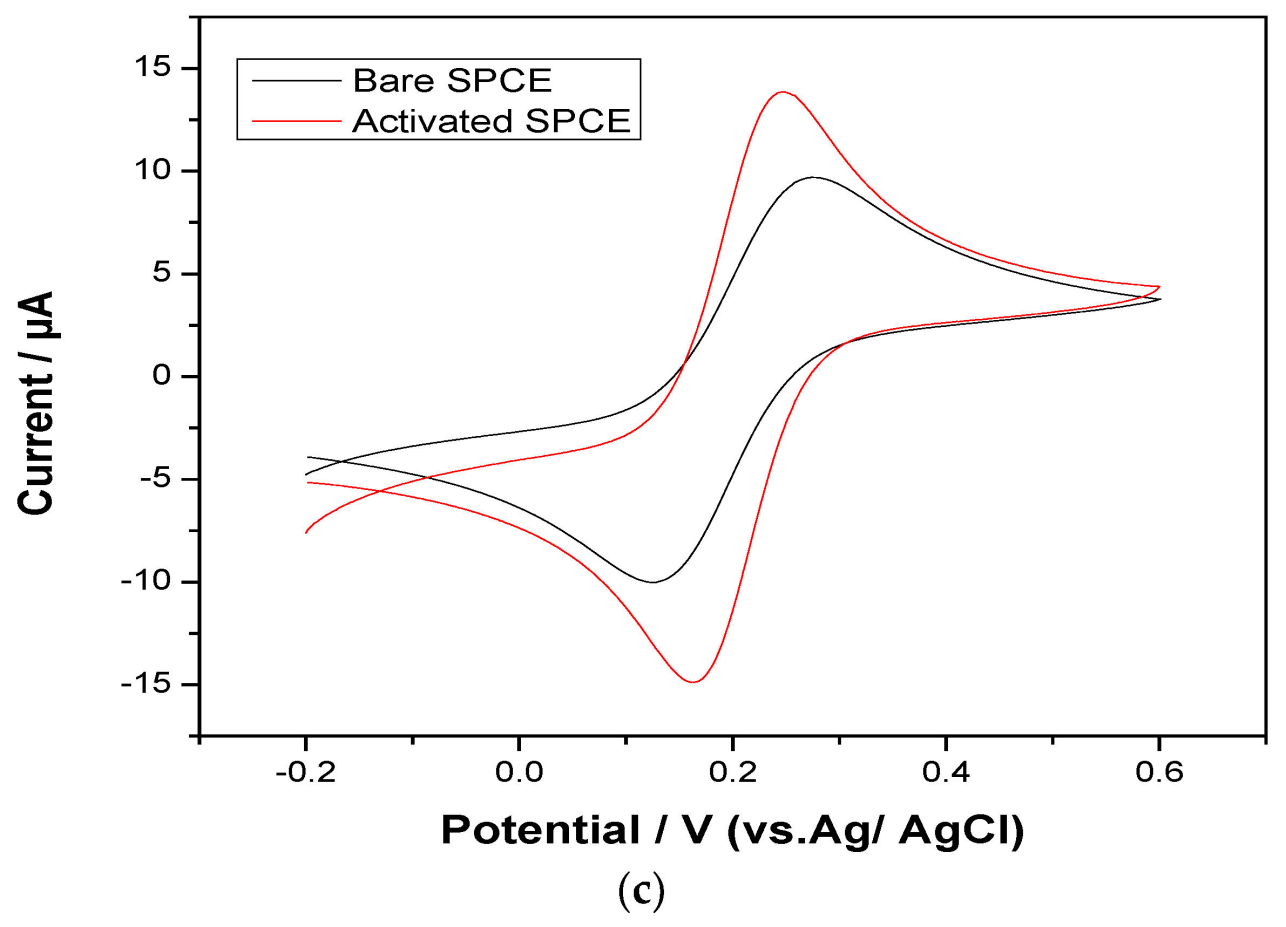
Cyclic voltammograms and plots of peak current vs. square root of scan rate (inset) of bare SPCE (**a**) and activated SPCE (**b**) with various scan rates from 20 mV/s to 180 mV/s. (**c**) The cyclic voltammograms of bare and activated SPCEs at 0.1 scan rate: in 1 mM K_3_[Fe(CN)_6_] in 0.1 M of KCl.

The surface of the screen-printed carbon electrode was activated to improve the electrode responses by cycling the electrode between 0 and 1.6 V in 0.5 M sulphuric acid at a 0.1 scan rate [42]. The CVs of the bare and activated electrodes are shown in Figure 2a,b. The electron transfer kinetics of the activated SPCE showed dramatic improvements. Moreover, a significant reduction in the separation between anodic and cathodic peak potential was reported for the treated electrode (from 186 mV to 145 mV, respectively), as well as a significant improvement in the current response from 9.83 µA to 14.97 µA at a 0.1 scan rate (Figure 2c). Obviously, it can be observed that there was a linear relationship between oxidation current and the square root of the scan rate, indicating that the reversible electron transfer reaction is a perfect, diffusion-controlled process, as seen in the Figure 2a,b insets. These enhancements can be attributed to increased surface hydrophilicity, an increase in the functional groups of carbon and oxygen on the surface and the elimination of surface contaminants [43,44]. Additionally, SEM images (Section 3.3) show the electrode surface morphology before and after electrochemical pre-treatments with 5000× magnification. The differences were obvious, as, when anodic polarization was applied in an acidic solution, the particles were slightly peeled off, and the surface of the electrode was changed to be dim, black and rough, which was more likely as a result of the elimination of organic binders [44,45].

### 3.2. Optimization of Nanocomposite Sensor for Carbaryl Detection

**Figure 3:**
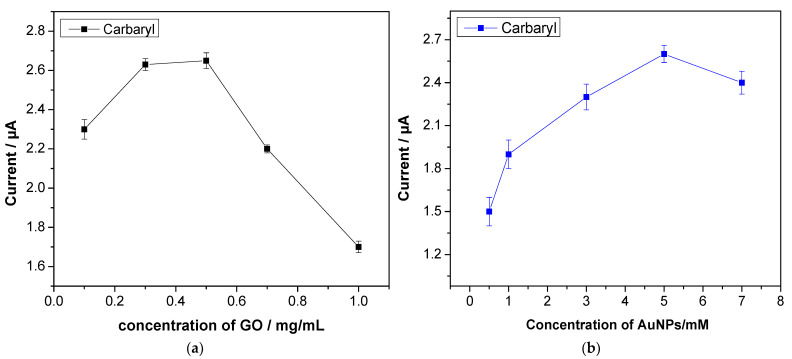

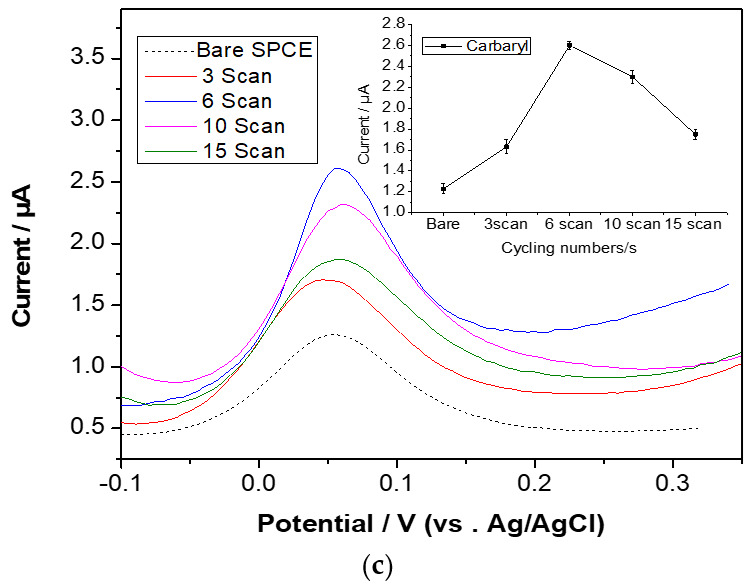
Effect of (**a**) quantity of GO in 5mM HAuCl4 solution and (**b**) concentration of AuNPs in 0.5 mg/mL of GO on 200 µM carbaryl current. (**c**) The effect of cycling numbers on the oxidation peak of 200 µM carbaryl; 0.1 M acetate buffer (pH = 5) at rGO/AuNP/NA/ SPCE.

The quantity of GO in 5mM HAuCl4 solution was evaluated in the range from 0.1 to 2.0 mg/mL with 200 µM carbaryl in 0.1 M acetate buffer (pH = 5). As seen in Figure 3a, the oxidation peak current increased gradually as the composite concentration increased from 0.1 to 0.5 mg/mL, achieving the highest current at 0.5 mg/mL. Then, as the concentration of GO was increased from 0.5 to 2.0 mg/mL, the oxidation peak current reduced due to the possibility that redundant rGO hampered electron transport between carbaryl and the electrode. The concentration of AuNPs was also varied from 0.5 mM to 10 mM in 0.5 mg/mL of GO. As seen in the figure, the carbaryl current increased with an increase in concentration from 0.5 mM to 5 mM and then the current response tended to be decreased (Figure 3b).

Additionally, the effect of cycling numbers on the reduction process on the carbaryl current response was investigated. Figure 3c illustrates that the oxidation peak current significantly improved when the cycling number increased from three to six scans. This is as a result of the majority of the surface area of the electrode, which was enhanced by the number of cycle reductions of the composite. However, the current was decreased by increasing the cycling number from 10 to 15 scans due to the size of AuNPs, which became larger and decreased the surface of the electrode [46]. Therefore, a 6-cycle of electro-reduction was selected for further optimisation.

### 3.3. Characterization of Activated and Nanocomposite-Modified SPC Electrodes

**Figure 4:**
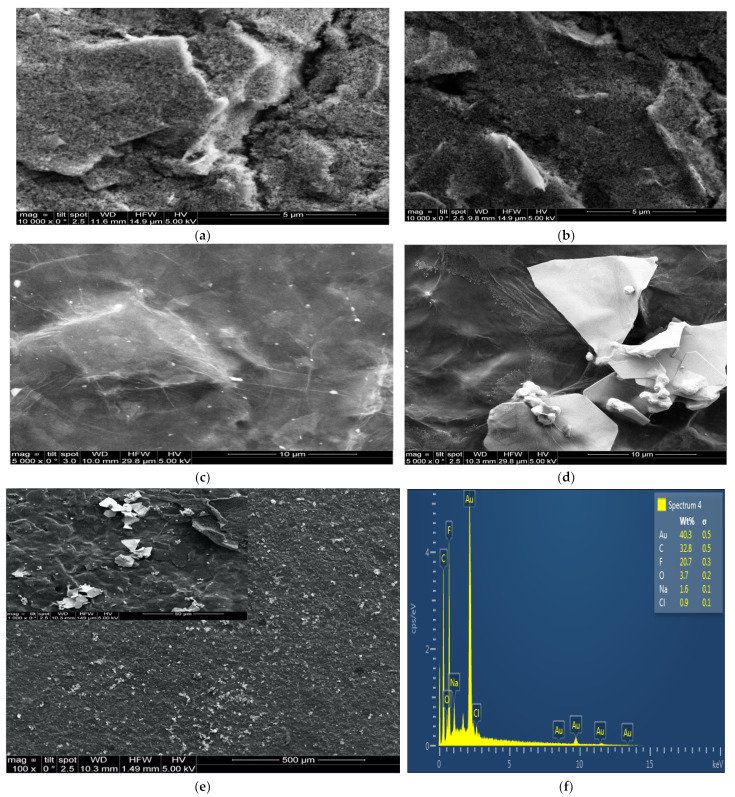
SEM images of bare (**a**), treated (**b**), rGO/NA (**c**) and rGO/AuNP/NA (**d**) SPCEs at 5000 magnification and (**e**) at 1000 magnification. EDX spectrum of rGO/AuNP/NA (**f**).

The surface morphology of the screen-printed carbon electrode was characterized using SEM. According to the SEM images in Figure 4a,b, the bare and activated electrodes showed non-uniform and heterogeneous graphite flakes of micrometric dimension separated from each other [47]. The graphene oxide nanosheets were accumulated on the electrode surface to form reduced graphene oxide, which appeared as typical, graphene-like sheet layers with wrinkles [48], as seen in Figure 4c. Simultaneously, the gold nanoparticles were then reduced on the reduced graphene oxide (rGO) nanosheets, presenting bright spheres distributed homogeneously onto the rGO sheets (Figure 4d) [49,50]. EDS analysis was also used to determine the elemental compositions, as shown in Figure 4f. The signal of O and F was attributed to Nafion, and C to rGO. The presence of AuNPs was confirmed by the signal of Au in the pattern. The elements of Na and Cl could be related to residual NaCl. Further analysis was performed using XPS to reveal information on the elemental composition and electronic state of the elements (Figure S1, Table S1).

**Figure 5:**
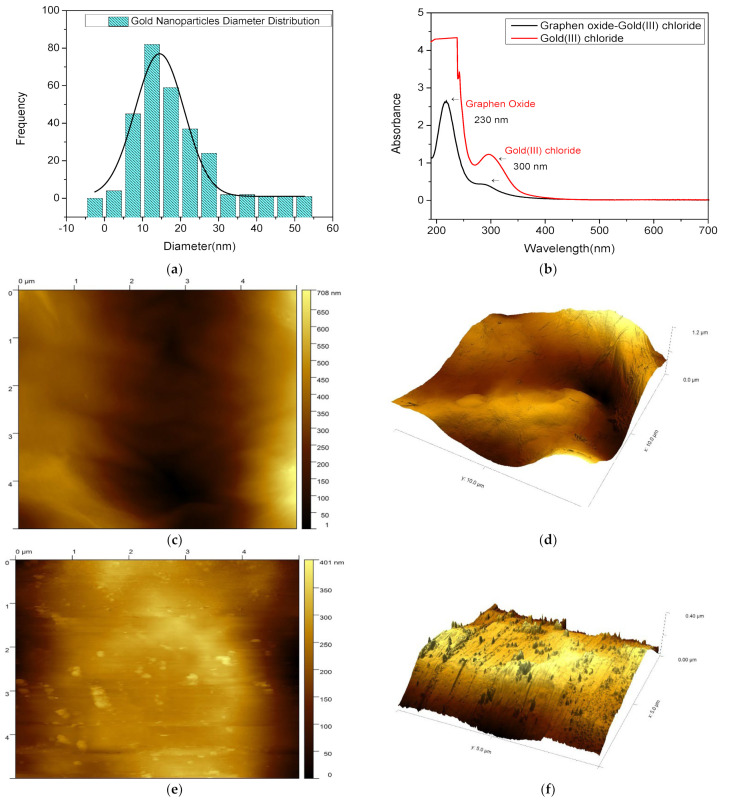
Particle size distribution histogram of gold nanoparticles(**a**). UV–vis spectra of graphene oxide and gold (III) chloride(**b**). Typical 2D (**c**) and 3D (**d**) AFM images of rGO/NA. Typical 2D (**e**) and 3D (**f**) AFM images of rGO/AuNP/NA.

To further characterize the modified electrode, AFM measurement were performed, as seen in Figure 5. The morphology of the gold nanoparticles demonstrated in the AFM images is consistent with the SEM micrographs. Figure 5e shows round gold nanoparticles that are irregularly dispersed on the substrate. Figure 4c shows the wrinkles and thin grooves on the surface of reduced graphene oxide. The AFM three-dimensional image shows more apparent wavy features (Figure 4d). Figure 4e shows unordered gold nanoparticles on the surface of the reduced graphene oxide sheets. The three-dimensional image (Figure 4f) shows the clear morphology of nanocomposites. The UV–visible range was used to characterize the absorption spectrum of the nanomaterials. As shown in Figure 5b, gold (III) chloride had a strong absorption maximum at a wavelength of 300 nm. Graphene oxide had an absorption peak centered at 230 nm.

**Figure 6:**
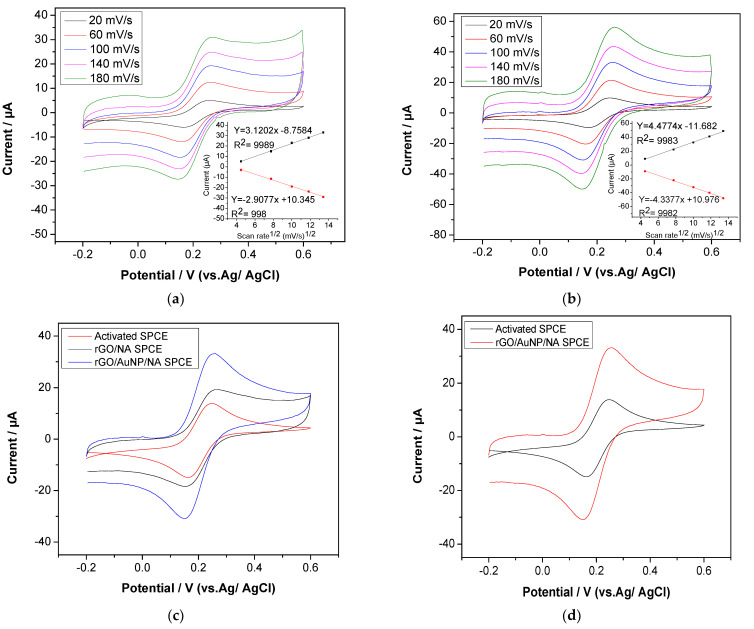
Cyclic voltammograms and plots of peak current vs. square root of scan rate (inset) for rGO/NA SPCE (**a**) and rGO/AuNP/NA SPCE (**b**) with various scan rates from 20 mV/s to 180 mV/s. (**c**) Cyclic voltammograms of activated, rGO/NA and rGO/AuNP/NA SPCEs at 0.1 scan rate. (**d**) Cyclic voltammograms of activated and rGO/AuNP/NA SPCEs at 0.1 scan rate: in 1 mM K_3_[F (CN)_6_] in 0.1 M of KCl.

The electrochemical behavior of different nanocomposite-modified electrodes was evaluated using cyclic voltammetry in the presence of 1 mM K_3_[Fe(CN)_6_] in 0.1 M of KCl solution at a scan rate of 0.1 mV/s. Figure 6c shows the anodic and cathodic peaks of the activated SPCE, rGO/NA SPCE and rGO/AuNP/NA SPCE with a potential difference (ΔE) of 145, 130 and 98 mV, respectively. This indicates that the lower the potential difference, the faster the electron transfer, which is due to the electrocatalytic behavior of the nanocomposite. Additionally, the rGO/NA SPCE and rGO/AuNP/NA SPCE exhibited considerably greater redox peak currents, suggesting higher electrochemical performance. This can be attributed to the electrocatalytic activity of the modified sensor, as well as the large effective surface area. Moreover, a higher-intensity current was obtained for the rGO/AuNP/NA SPCE with a better redox behavior, demonstrating that the combination of rGO and AuNPs enhances the electroactive surface and further accelerates electron transfer.

Cyclic voltammetry was also used to evaluate the reversibility and diffusion-controlled properties of the redox probe. For modified nanocomposite electrodes, plots of peak current vs. the square root of the scan rate are displayed in Figure 6a,b (inset). Based on these figures, the peak currents and the square root of the scan rate values indicate a perfect linear relationship, showing that the reversible electron transfer reaction is completely diffusion controlled. Moreover, to evaluate the sensitivity of detection, cyclic voltammetry was used to measure the modified nanocomposite sensor’s effective electroactive surface area using the Randles–Sevcik equation:

I_p_ = (2.69 × 10^5^)C_O_n^3/2^Y^1/2^D^1/2^A (1)

where I_p_ represents the anodic peak current in amperes, C_O_ the redox probe concentration in M, n the number of transferred electrons (n = 1), D the diffusion coefficient in cm^2^/s, v the scan rate in mV/s and A the electrode surface area in cm^2^. From these figures, according to the fitted curve of I_p_ vs. v^1/2^ for the activated SPCE, rGO/NA SPCE and rGO/AuNP/NA SPCE, the effective electroactive surface area was determined to be 0.30 cm^2^, 0.42 cm^2^ and 0.59 cm^2^, respectively. As demonstrated, the A value of rGO/NA SPCE was approximately 39.8% higher than the activated, screen-printed carbon electrode, which can be attributed to the high conductivity of rGO and large surface area of the electrode. Furthermore, the electrode’s electroactive surface area rose by approximately 42.9% after being modified with nanocomposites of rGO and AuNPs, owing to the synergistic impact of the conductivity between rGO and the AuNPs.

**Figure 7:**
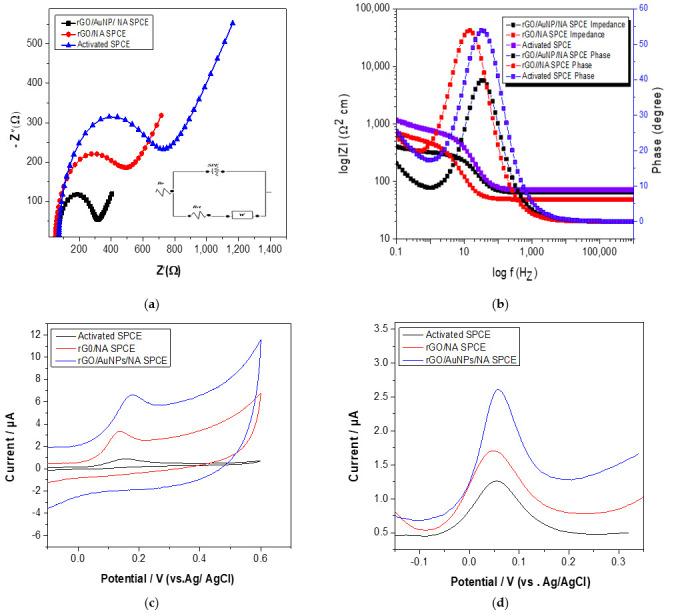
Nyquist plots (**a**,**b**) bode plots of EIS for activated SPCE, rGO/NA SPCE and rGO/AuNP/NA SPCE using 1 mM K_3_[Fe(CN)_6_] in 0.1 M of KCl. Cyclic voltammograms (**c**) and DPV (**d**) of 200 µM carbaryl in 0.1 M acetate buffer (pH = 5) at activated SPCE, rGO/NA SPCE and rGO/AuNP/NA SPCE.

In terms of impedance changes, to evaluate the interface properties of the nanocomposite-modified electrodes, electrical impedance spectroscopy (EIS) was employed (the EIS parameters are shown in Figure S2). The diameters of the semicircles are known to be comparable to the resistances to the faradaic charge transfer (R_ct_) of the modified electrodes [51]. Consequently, the semicircle diameters were used to determine the order of the R_ct_ of several nanocomposite sensors. As shown in Table 1, by comparison, the greatest semicircle was detected in the activated SPCE EIS plot, indicating a slower interfacial charge transfer. Obviously, the R_ct_ was dramatically reduced by 40% at the rGO-modified carbon electrode. The lower R_ct_ value over the modified electrodes can be attributed to the nanocomposite’s superior electrical conductivity, which can enhance the electron transfer and mass exchange of electroactive indicators on the surface. The results of Nyquist and bode plots of EIS for activated and modified SPCEs are shown in Figure 7a,b. The analysis results of EIS are consistent with cyclic voltammogram studies.

Further characterization using electrochemical impedance spectroscopy was performed to investigate the charge transport process of the rGO/NA SPCE and rGO/AuNP/NA-modified, screen-printed carbon electrodes. The electron transfer rate constant (K_et_) values were calculated using Equation (2), as shown below:

K_et_ = RT/n^2^F^2^AC_0_R_ct_ (2)

where R is the gas constant (8.314 J/K mol), T is the temperature in Kelvin degrees, n is the number of electrons, F is Faraday’s constant (96,485 C/mol), A is the electrode surface area in cm^2^, C is the redox probe concentration in mol/L and R_ct_ is the charge transfer resistance in Ω, which is determined based on impedance measurements. Using Equation (2), the electron transfer rate constant values for activated and modified electrodes were calculated, as shown in Table 1. The K_et_ value of the rGO/AuNP/NA-modified electrode was higher than those of the rGO-modified and activated, screen-printed electrodes, indicating a higher rate of electron transfer between the rGO/AuNP/NA-modified SPCE interface and redox species, which was consistent with the E values.

Based on the presented results, it is possible to conclude that the electrochemical performance of the modified rGO/AuNP/NA SPCE was developed. This can be attributed to the nanocomposite’s synergistic properties, which provide a wide active surface area and a high rate of electron transfer. The modified rGO/AuNP/NA SPCE was analyzed using cyclic voltammetry and DPV in a solution containing 200 µM carbaryl–phenol in 0.1 M acetate buffer (pH = 5). The recorded voltammograms are shown in Figure 7c,d, presenting a higher oxidation current for the modified nanocomposite compared to the bare SPCE. It was approximately 100% higher than the naked electrode. These findings indicate that rGO and AuNPs are both valuable materials that are capable of increasing the sensitivity of the electrochemical detection of carbaryl.

### 3.4. Optimization Study

Effect of pH

**Figure 8:**
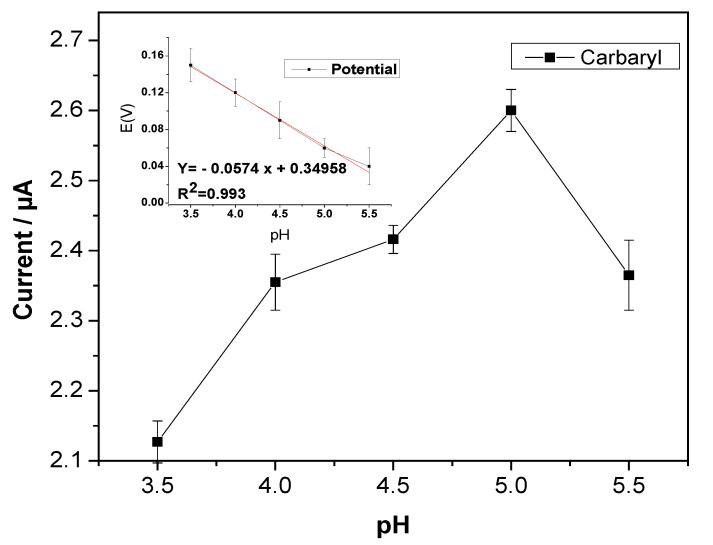
The effect of pH of 0.1 M sodium acetate buffer on the oxidation current and potential of 200 µM carbaryl at rGO/AuNP/NA SPCE.

The influence of pH on the peak potential and current of carbaryl is significant. Therefore, the electrochemical performance of carbaryl was investigated in acetate buffer within a pH ranging from 3 to 5.5. According to Figure 8, with increasing pH, the potential moved to more negative values, indicating the linear relationship between pH and- current potential. Additionally, the equation was determined to be Ep/V = 3.4958 − 0.0574 pH with an R value equal to 0.0993, and the slope was 57 mv pH per unit, which was nearly equal to the theorical value (59 mv) as per Nernst’s theoretical equation. As a result, the carbaryl oxidation process involved an equal number of protons and electrons. It was obvious that, at a pH of 5, the current achieved its highest value and then tended to decline, which could be due to the molecule protonation and the production of naphthoxide species [14,51]. pH 5 was selected as the effective pH for the following research. 

### 3.5. Calibration, Selectivity, Stability and Reproducibility

**Figure 9:**
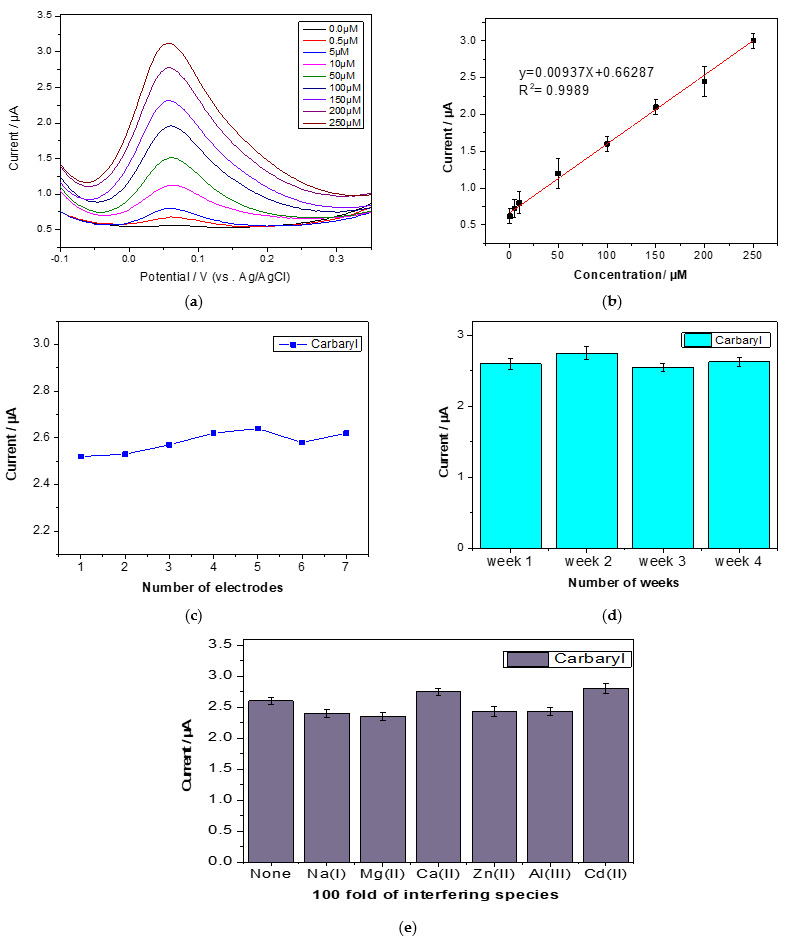
The voltammograms (**a**) and calibration (**b**) of carbaryl–phenol in 0.1 M acetate buffer (pH = 5) using rGO/AuNP/NA SPCE. The effect of (**c**) reproducibility, (**d**) stability and (**e**) selectivity on the current of carbaryl–phenol in 0.1 M acetate buffer (pH = 5) using rGO/AuNP/NA SPCE.

Under optimum conditions, DPV was used to determine carbaryl–phenol concentrations ranging from 0.5 µM to 250 µM. Figure 9a shows the resulting voltammograms with various carbaryl–phenol concentrations. The peak currents obviously increased with the increasing concentrations, indicating very good linearity with a correlation coefficient of 0.9989 (Figure 9b). The calibration equation of carbaryl was y = 0.0094X + 0.663 with a sensitivity of 0.01 µA/µM. The limit of detection and quantification was estimated to be 0.2 and 3.5, respectively. The reproducibility of the modified, screen-printed carbon electrode was evaluated using seven electrodes, which were prepared in the same manner. The relative standard deviation (RSD) of 200 µM carbaryl was 1.7%, indicating a very high degree of reproducibility (Figure 9c). Additionally, the developed electrode stability was studied, and it was discovered that the electrode could be used for a month with a slight decrease in the carbaryl oxidation peak current under the ideal condition (Figure 9d). Furthermore, under the optimized conditions, the interference study was investigated in the presence of a variety of possible interfering species in 0.1M acetic buffer containing 200 µM carbaryl. The 100-fold Ca^2+^, Mg^2+^, Na^+^, Zn^2+^, Cd^2+^ and Al^3+^ did not significantly affect the determination of carbaryl. It was observed that the effect of these species on the oxidation current of carbaryl was approximately 10% (Figure 9e). The DPV of carbaryl–phenol in the presence of interfering ions is shown in Figure S3.

### 3.6. Real Samples Analysis

The developed electrochemical screening assay for the determination of carbaryl was applied to real tap and river water samples. Using the standard addition method, the samples spiked with carbaryl standard were analyzed, and the results are shown in Table 2. The developed method had good accuracy, with recovery values in the range of 88.6% to 111.6% and RSDs ≤ 1.67%. In comparison, the result obtained by rGO/AuNP/NA assay was compared with the conventional CE-UV method. Table 2 shows the extraction recoveries for carbaryl with recovery values (in the 96–125.5% range) and good reproducibility (RSDs ≤ 1.6%, n = 3 extraction replicates). It should be noted that the developed sensor, using hydrolysis and electrochemical measurement, was able to determine carbaryl at a lower detection limit than traditional CE, the concentration ranges of which are between 60 and 1000 µM, indicating that the developed sensor is highly sensitive and easy to use for carbaryl determination. A comparison between the electrochemical performance of various developed electrodes for carbaryl determination using direct and indirect techniques is summarized in Table 3. The obtained sensor had a satisfactory result, which is promising for simple and low-cost analysis in real fields and has the potential to be integrated as an on-site monitoring system for environmental water.

## 4. Conclusions

In this study, an inexpensive and effective method based on electrochemical deposition for the electrochemical determination of carbaryl in water samples was developed. A simple, single-step synthesis of rGO/AuNP/Nafion showed excellent electrochemical performance with a limit of detection and quantification of 0.2 µM and 3.5 µM, respectively. The surface morphology of the screen-printed carbon electrode was characterized using scanning electron microscopy (SEM), atomic force microscopy (AFM), dispersive X-ray spectroscopy (EDX) and X-ray photoelectron spectroscopy (*XPS*) analysis. Further characterization using electrochemical electrical impedance spectroscopy (EIS) and cyclic voltammetry was performed to investigate the electrochemical behavior of different nanocomposite-modified electrodes. Under optimum conditions, DPV was used to measure carbaryl–phenol concentrations ranging from 0.5 µM to 250 µM with a correlation coefficient of 0.9989. Additionally, the modified SPCE offers various advantages over previous electrochemical sensors, including ease of fabrication, high efficiency, outstanding reproducibility, great stability and high selectivity. The effect of interfering ions was slight on the oxidation current of carbaryl–phenol, and the presented assay showed excellent stability and great reproducibility with a relative standard deviation of 1.7%. The developed sensor was successfully applied in river and tap water samples and compared with the traditional CE-UV method, indicating good accuracy with recovery values in the range of 88.6% to 111.6% and RSDs ≤ 1.67%.

## Figures and Tables

**Table 1:** The values of charge transfer resistance (R_ct_) and rate constant (K_et_) for bare and modified electrodes.

Electrode	Charge Transfer Resistance (R_ct_) (Ω)	Rate Constant (K_et_) (cm^2^)
Activated SPCE	0.7 × 10^2^	1.1 × 10^−1^
rGO/NA/SPCE	0.5 × 10^2^	1.2 × 10^−1^
rGO/AuNPs/NA/SPCE	0.3 × 10^2^	1.3 × 10^−1^

**Table 2:** Carbaryl concentration in spiked samples using the developed rGO/AuNPs/NA/SPCE and traditional CE.

Sample	Spiked (µM)	rGO/AuNPs/NA/SPCE			CE-UV		
		Found	Recovery%	RSD%	Found	Recovery%	RSD%
River water	60.00	53.30	88.60	2.30	75.3	125.5	1.60
	125.00	115.56	92.45	3.10	145.3	116.24	2.5
Tap water	60.00	67.00	111.6	2.67	59.00	98.30	1.75
	125.00	120	96	2.25	120	96	1.70

**Table 3:** Electrochemical analytical performance based on nanocomposites sensor for carbaryl detection.

Electrode	Analyte (Carbaryl)	Linear Range (µM)	LOD (µM)	Sample	Ref.
Poly-pPDs IL/CPE	Indirect	0.5–200	0.09	water and fruit	[52]
Carbon black nanoparticles/SPE	Indirect	0.1–100	0.048	food	[53]
CoO/rGO/GCE	Indirect	0.5–200	7.5	fruit and vegetables	[54]
GO-IL/GCE	Indirect	0.1–12	0.02	fruit	[55]
Low silica X zeolite modified/CPE	Indirect	1–100	0.3	tomato	[56]
MWCNT/CoPc/GCE	Direct	0.30−6.61	0.005	river water	[14]
GR/BDD	Direct	1–6	0.07	apple juice	[57]
rGO/AuNP/NA/SPCE	Indirect	0.5–250	0.2	river and tap water	This work

Poly-pPD: poly–poly p phenylenediamine, CPE: carbon paste electrode, CPE: carbon paste electrode, SPE: screen-printed carbon electrode. CoO: cobalt (II) oxide, rGO: reduced graphene oxide, GCE: glassy carbon electrode, GO: graphene oxide, IL: ionic liquid, AuNPs: gold nanoparticles, SPCE: screen-printed carbon electrode. CoPc: cobalt phthalocyanine, MWCNT: multi-wall carbon nanotubes. GR: graphene, BDD: doped diamond electrode.

## Data Availability

Data are contained within the article.

## Supplementary Materials

The following supporting information can be downloaded at: https://www.mdpi.com/article/10.3390/s22145251/s1, Figure S1: XPS Analysis of rGO/AuNP/NA SPCE; the signal of Au (a), C (b), O (c) and F (d); Table S1: Quantification from high resolution spectra for rGO/AuNP/NA SPCE surface; Figure S2: The equivalent circuit of impedance spectra and EIS parameters; Figure S3: DPV of 200 μM carbaryl-phenol in 0.1 M acetate buffer (pH = 5) in the presence of 100-fold Ca2+, Mg2+, Na+, Zn^2+^, Cd^2+^, and Al^3+^ using rGO/AuNP/NA SPCE.
