# Supplementary material for: Electrochemical Synthesis of Reduced Graphene Oxide/Gold Nanoparticles in a Single Step for Carbaryl Detection in Water
Source: Sensors (Basel). 2022 Jul 13;22(14):5251. doi: 10.3390/s22145251 (PMC9317711; doi:10.3390/s22145251)
Supplement: Supplementary file 1 [file sensors-22-05251-s001.zip › sensors-1766621-supplementary.pdf]

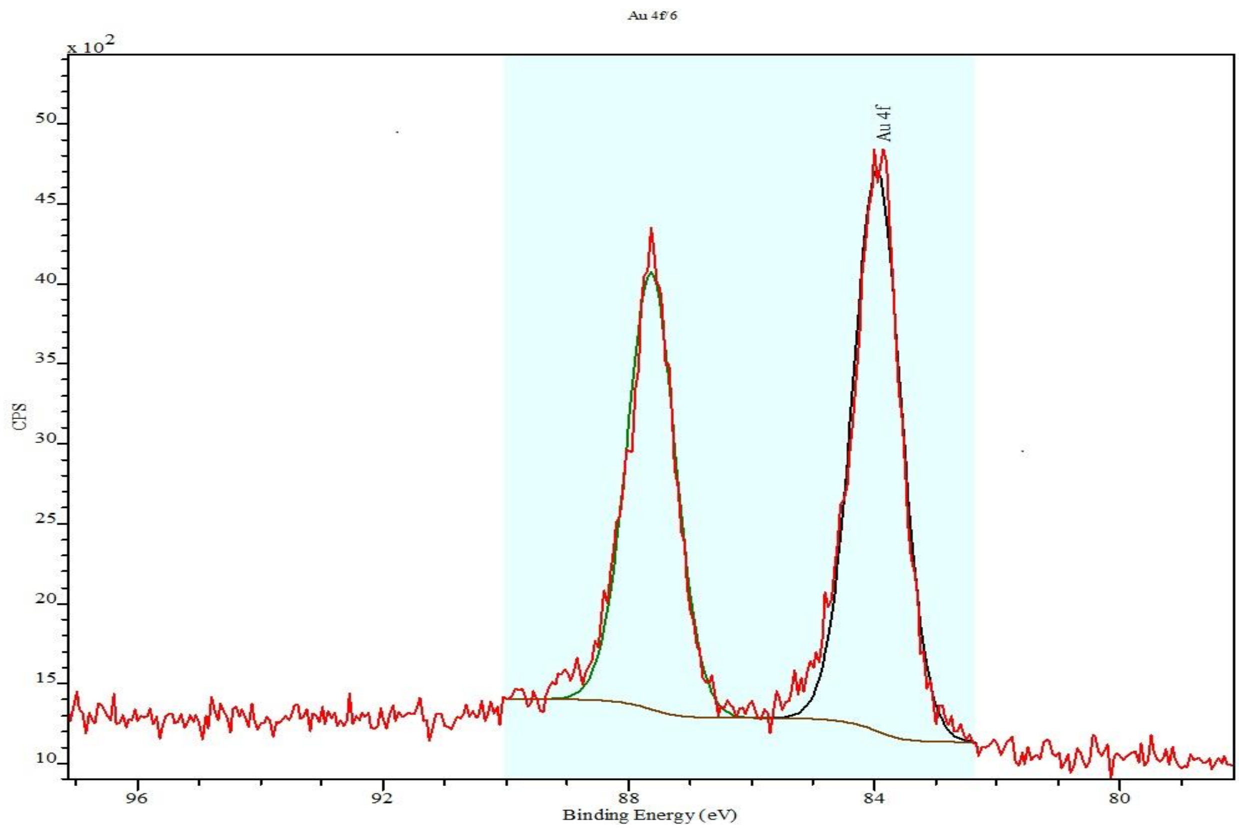
Au 4f 6
x 10^2
50
45
40
35
30
25
20
15
10
CPS
Au 4f
96
92
88
84
80
Binding Energy (eV)

(a)

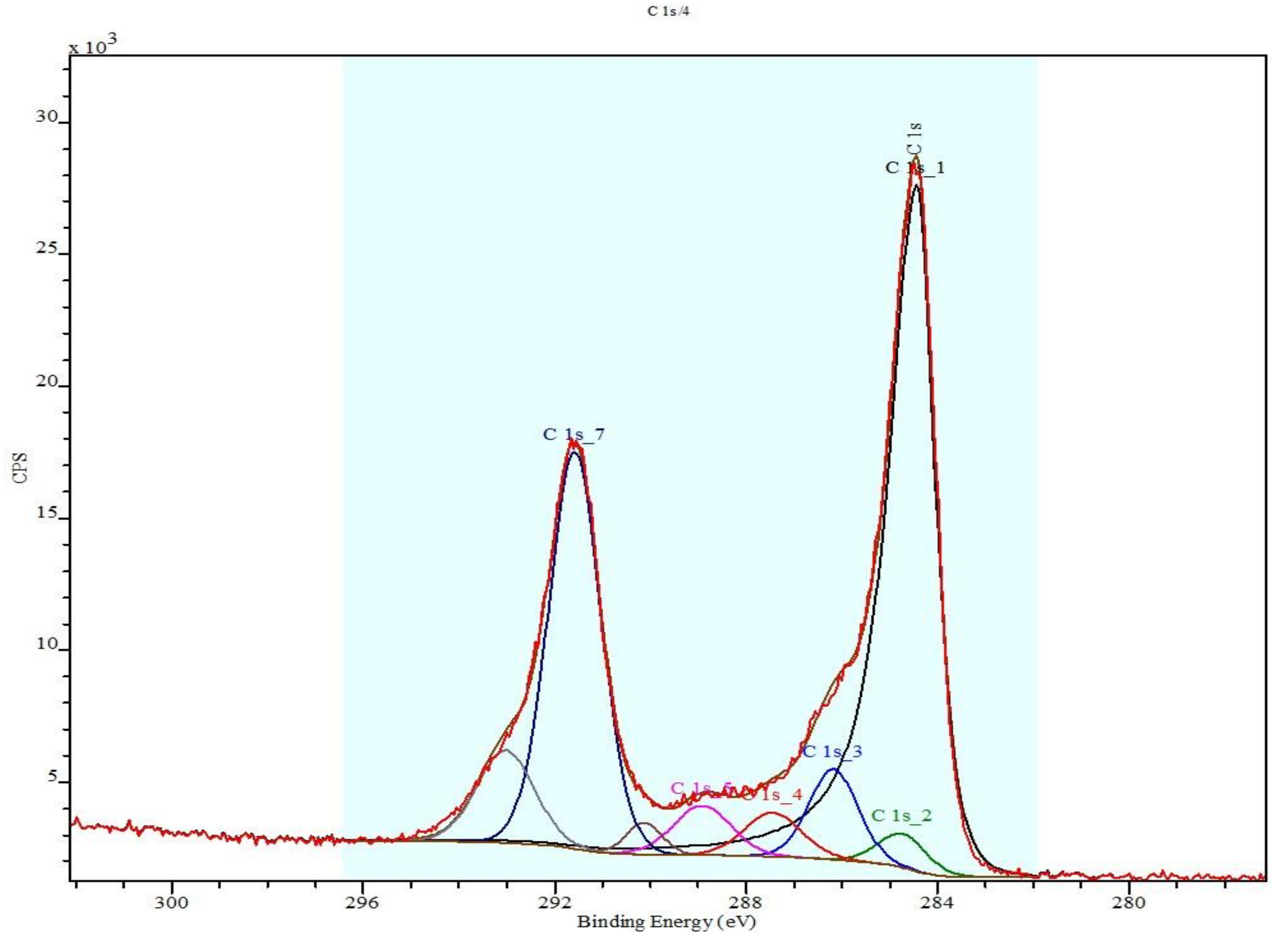

C 1s/4
x 10^3
30
25
20
15
10
5
CPS
C 1s
C 1s_1
C 1s_7
C 1s_5
C 1s_4
C 1s_3
C 1s_2
300
296
292
288
284
280
Binding Energy (eV)

(b)

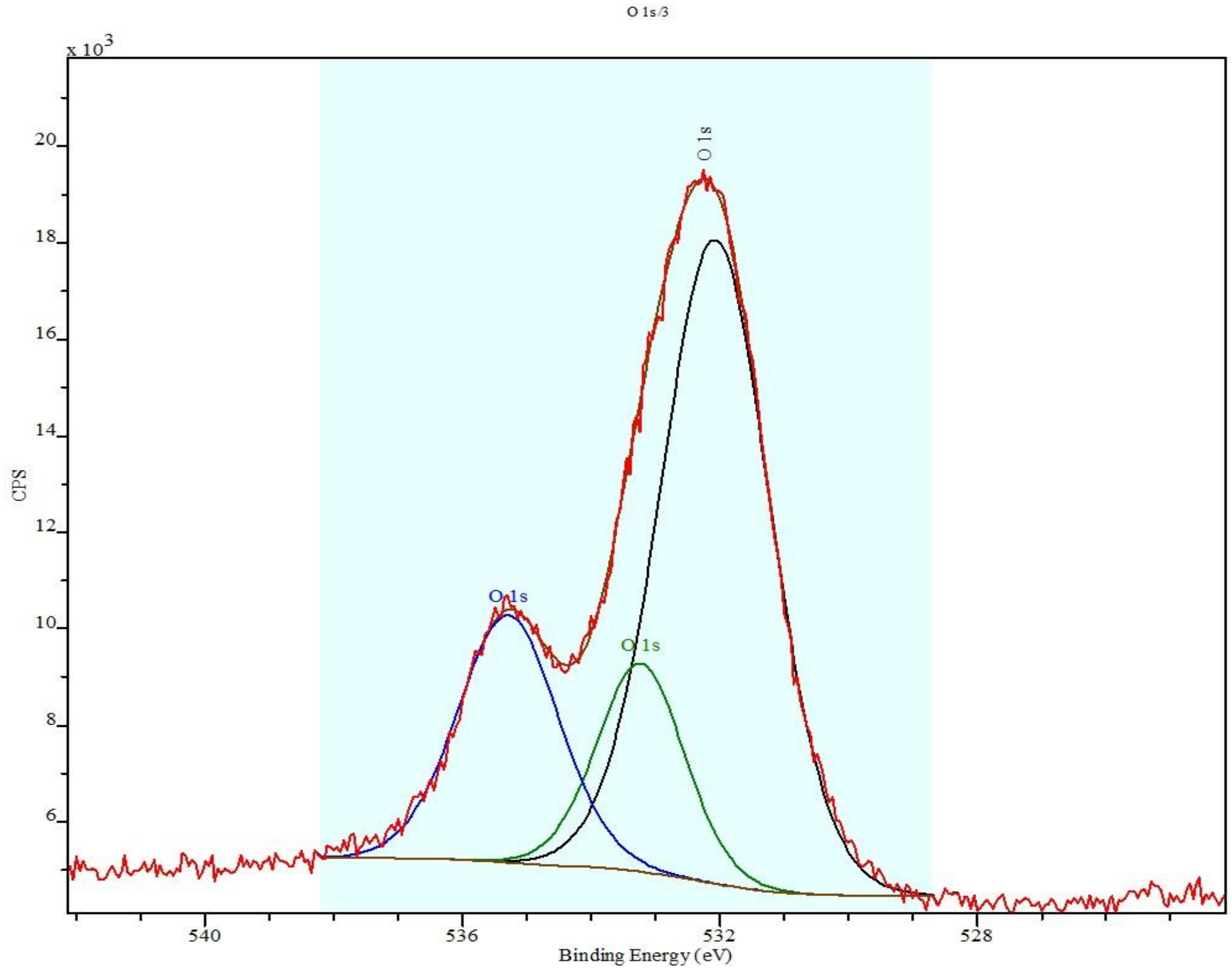
O 1s/3
x 10^3
20
18
16
14
12
10
8
6
CPS
O 1s
O 1s
O 1s
540
536
532
528
Binding Energy (eV)

(c)

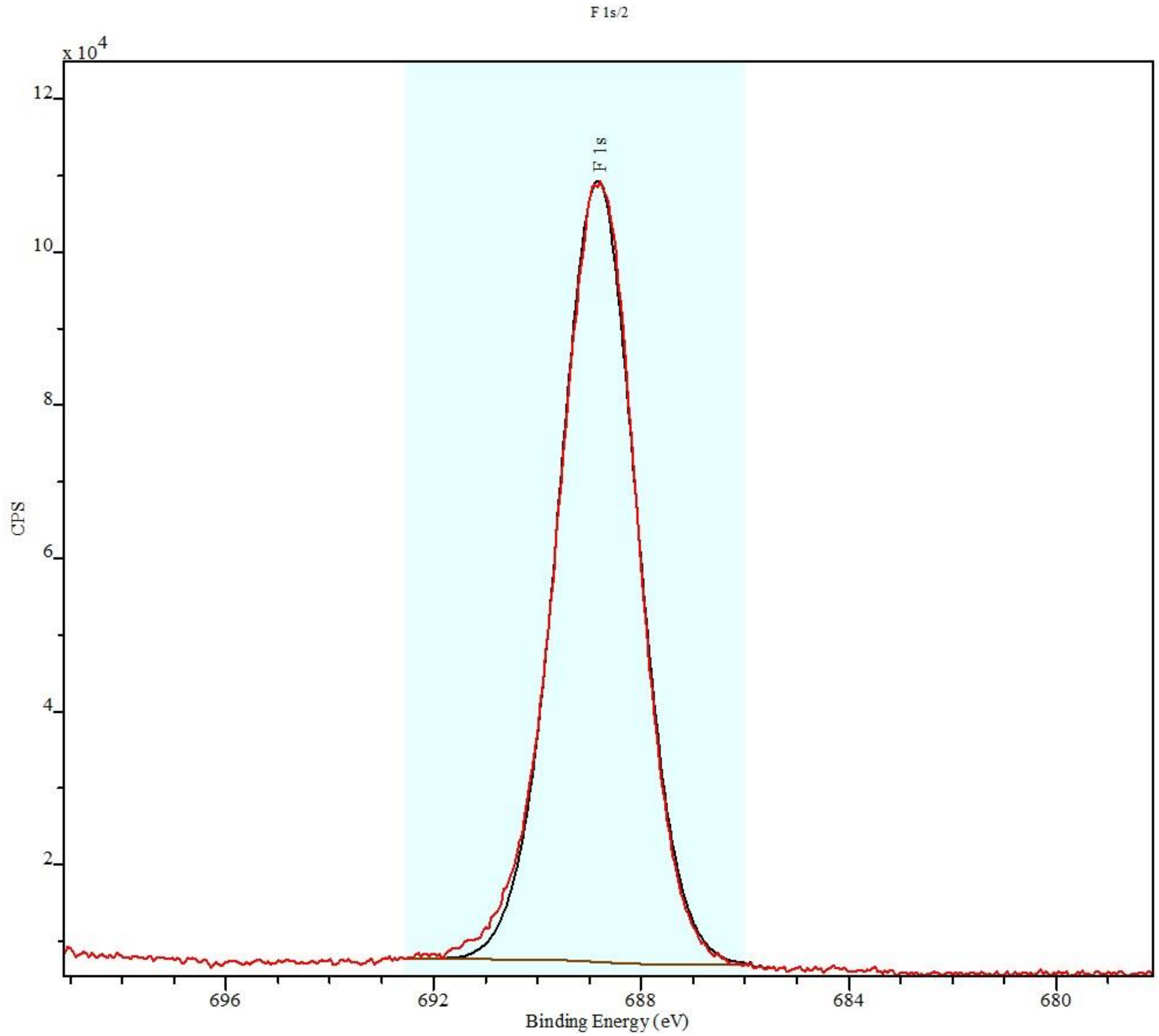

(d)

Figure S1. XPS Analysis of rGO/AuNP/NA SPCE ; the signal of Au (a), C (b) , O (c) and F (d). Area is approximately 1 mm^2.

Name	Position	FWHM	R.S.F.	Area	% Conc.	
F 1s	688.8	1.7	4.43	214232	34.9	
O 1s	532.3	2.4	2.93	48507	11.9	
C 1s	284.5	1.2	1	71247	51.3	
Au 4f	184.5	0.9	2.1	65564	41.2	
F 1s	688.8	1.7	4.43	212225	34.9	C-F
O 1s_1	532.1	2.0	2.93	29912	7.4	O from organics ,
O 1s_2	533.2	1.6	2.93	7702	1.9	C-O
O 1s_3	535.3	1.8	2.93	10564	2.6	
C 1s_1	284.5	0.9	1	35891	25.7	C=C
C 1s_2	284.8	1.2	1	1495	1.1	C-C
C 1s_3	286.2	1.2	1	4101	2.9	C-O
C 1s_4	287.4	1.4	1	2350	1.7	C=O
C 1s_5	288.9	1.4	1	2632	1.9	O-C=O
C 1s_6	290.1	0.9	1	1022	0.7	pi to pi* satellite
C 1s_7	291.6	1.3	1	19320	13.8	CF2
C 1s_8	293.0	1.4	1	4917	3.5	CF3
Au 4f_1	84.0	0.9	17.1	2998	0.13	4f7/2: Au(0)
Au 4f	87.6	0.9	17.1	2341	0.10	4f5/2

Table S1. Quantification from high resolution spectra for rGO/AuNP/NA SPCE surface.

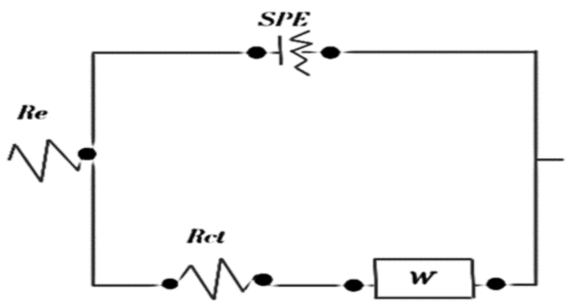

Impedance parameters and fitted circuit:
Init E (V) = 0.2 for activated SPCE,
Init E (V) = 0.35 for rGO/NA
Init E (V) =0.4 for rGO/AuNPs/NA
High Frequency (Hz) = 1e+6
Low Frequency (Hz) = 0.1
Amplitude (V) = 0.1
Quiet Time (sec) = 5
Cycles (.1-1Hz) = 1

Figure S2. The equivalent circuit of impedance spectra and EIS parameters.

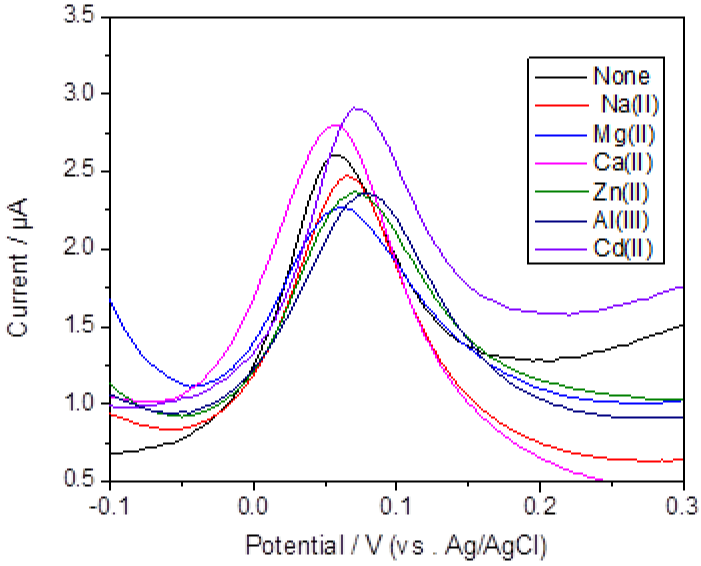

Figure S3. DPV of 200 µM carbaryl -phenol in 0.1 M acetate buffer (pH=5) in the presence of 100-fold Ca^{2+}, Mg^{2+}, Na^{+}, Zn^{2+}, Cd^{2+}, and Al^{3+} using rGO/AuNP/NA SPCE .
